# An Ultra-High Performance Liquid Chromatography with Tandem Mass Spectrometry Method for Determination of 10 Alkaloids in Beagle Dog Plasma after the Oral Administration of the *Corydalis yanhusuo* W.T. Wang Extract and Yuanhuzhitong Tablets

**DOI:** 10.3390/molecules23081925

**Published:** 2018-08-02

**Authors:** Binbin Cui, Jing Yang, Zhibin Wang, Chengcui Wu, Hongrui Dong, Yixuan Ren, Chunjuan Yang

**Affiliations:** 1 Department of Pharmaceutical Analysis and Analytical Chemistry, College of Pharmacy, Harbin Medical University, No. 157 Baojian Road, Nangang District, Harbin 150081, Heilongjiang, China; binbincui0419@163.com (B.C.), 18845645250@163.com (C.W.); donghongrui422@163.com (H.D.); renyixuan1218@163.com (Y.R.); 2 Department of Physiology, Basic Medical College of Heilongjiang University of Chinese Medicine, Harbin 150040, Heilongjiang, China; yangjingdx@sina.com; 3 Key Laboratory of Chinese Materia Medica (Ministry of Education), Heilongjiang University of Chinese Medicine, Harbin 150040, Heilongjiang, China; wzbmailbox@126.com

**Keywords:** ultra-high performance liquid chromatography-electrospray ionization-tandem mass spectrometry, *Corydalis yanhusuo* W.T. Wang, Yuanhuzhitong tablets, pharmacokinetics

## Abstract

This study has developed a sensitive and simple ultra-high performance liquid chromatography-electrospray ionization-tandem mass spectrometry method for the simultaneous determination of corydaline, dehydrocorydaline, tetrahydropalmatine, protopine, palmatine, tetrahydroberberine, columbamine, berberine, coptisine and berberrubine in beagle dog plasma after the oral administration of the *Corydalis yanhusuo* W.T. Wang and Yuanhuzhitong tablets. Chromatographic separation was achieved on an Agilent Eclipse Plus C18 RRHD column (1.8 µm, 50 × 2.1 mm) using a gradient elution program with a mobile phase consisting of acetonitrile and water containing 0.1% formic acid at a flow rate of 0.3 mL/min. A tandem mass spectrometric detection was conducted by multiple reaction monitoring (MRM) mode via an electrospray ionization source in the positive mode. The calibration curves of all analytes showed good linear (*r*^2^ > 0.9800). The intra-day and inter-day precisions were less than 15% and the accuracies were within ±15%. The extraction recoveries conformed to the acceptable range. And there was no interference of endogenous substances in the sensitive assay method. All analytes were proven to be stable during sample storage and analysis procedures. The pharmacokinetic study indicated that the Yuanhuzhitong tablets could get a better absorption than *Corydalis yanhusuo* W.T. Wang.

## 1. Introduction

Yuanhuzhitong tablet (YHZT), formally incorporated into Chinese Pharmacopoeia and Chinese National Essential Medicine List, consists of two herbs including 223 g of Radix Angelicae dahuricae and 445 g of vinegar-processed *Corydalis yanhusuo* W.T. Wang (*C. yanhusuo*) [1]. As a classic Chinese Patent Drug, YHZT has been frequently applied to alleviate various pains including hypochondriac pain, headache, stomachache and dysmenorrhea [1]. Chemically, alkaloids and coumarins are the main active constituents of YHZT, which possess a variety of pharmacological actions, such as analgesia, spasmolysis, anti-inflammatory, antianxiety and vasodilatation [2]. The vinegar-quenching processing technology has been utilized for the extraction of the active ingredient of *C. yanhusuo* due to a better solubility and to increase the curative effect [3]. The processing method of vinegar *C. yanhusuo* has a long application history in traditional Chinese medicines (TCM). Previous study showed that the alkaloid content of *C. yanhusuo* was higher with vinegar in its decoction process than that without it [4]. TCM is often composed of two or more herbs to obtain synergistic effects or to reduce possible adverse reactions [5,6,7]. Clinical practice and previous pharmacological studies have proven that Radix Angelicae dahuricae could enhance the analgesic effect of *C. yanhusuo* [8].

*C. yanhusuo*, which is also called *yuanhu* in China, is a key constituent herb in YHZT. It is one of the most significant dried medicinal herbs used in TCM [9]. *C. yanhusuo* was recorded in the Pharmacopoeia of the People’s Republic of China in 2015 [1]. It has been used for centuries for analgesic, sedation, anti-arrhythmic. It has been studied more and more nowadays since the recent discovery of its cancer pain relief effect [10,11,12,13,14]. A number of its constituents have been isolated and reported, including alkaloids, aliphatic acid, ecdysterone, etc [15]. Alkaloids were identified as the main effective components in *C. yanhusuo* according to the pharmacological study results. Meanwhile, tetrahydropalmatine is the one alkaloid that has been selected as a phytochemical marker for the quality control of *C. yanhusuo* in the Chinese Pharmacopoeia [1]. Tetrahydropalmatine, corydaline, tetrahydrobeberine and berberine have a significant analgesic effect [16,17]. Dehydrocorydaline and coptisine have the effect of anti-inflammatory and anti-coronary heart disease [18]. Tetrahydrobeberine have protection on cardiac cerebral vessel and antipsychotic effects [19]. Prptopine exhibits significant antihepatotoxic effects [20]. Palmatine, berberine and berberrubine are reported to possess antibacterial activity [21].

For a better understanding of pharmacokinetic behavior of alkaloids in the animal and human body, a sensitive analytical method needs to be established to quantitatively analyze the alkaloids in *C. yanhusuo* and YHZT. Several methods have been reported, including high-performance liquid chromatography with ultra-violet detection (HPLC-UV) [22,23], high-performance liquid chromatography with DAD detection (HPLC-DAD) [24], and high-performance liquid chromatography electrospray ionization-tandem mass spectrometry (HPLC-ESI-MS/MS) [25] for quantitative analysis of alkaloids in *C. yanhusuo* extract. The effect of the co-administration of *C. yanhusuo* and Radix *Angelicae dahuricae* on pharmacokinetic behavior of tetrahydropalmatine was investigated and the result showed that the plasma concentration of tetrahydropalmatine was higher in the combination pair group than that in the *C. yanhusuo* single herb group [26]. Many pharmacokinetic studies have been reported about the alkaloids from *C. yanhusuo* extract in rat plasma [27,28,29].

In this article, a new UHPLC-ESI-MS/MS method has been developed for simultaneous analysis of 10 alkaloids in beagle dog plasma after the oral administration of *C. yanhusuo* extraction and YHZT. It is the first article that analyse 10 alkaloids from *C. yanhuso* in biological fluids in a single run. According to the calculation, the dosage of *C. yanhusuo* extract and YHZT for beagle dog could be confirmed accurately. In the fasting state, the beagle dogs were given the equal amount of the two medicines. The absorption of alkaloids in beagle dog plasma was compared and their pharmacokinetic characteristics were elucidated, which would hopefully establish the basic groundwork for the further study and clinical application of *C. yanhusuo* and YHZT.

## 2. Results

### 2.1. Optimization of UHPLC-ESI-MS/MS Condition

**Figure 1:**
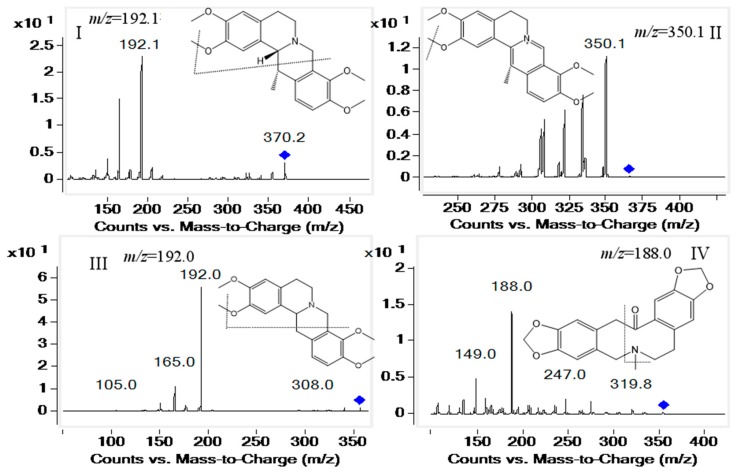

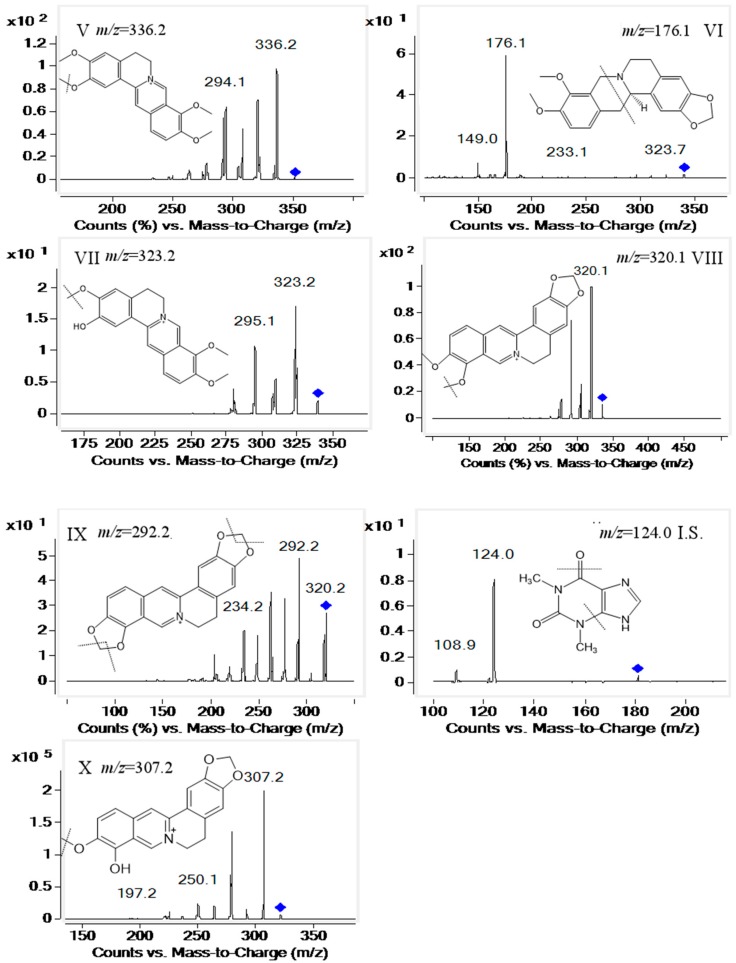
Product ion mass spectot of corydaline (**I**), dehydrocorydaline (**II**), tetrahydropalmatine (**III**), protopine (**IV**), palmatine (**V**), tetrahydroberberine (**VI**), columbamine (**VII**), berberine (**VIII**), coptisine (**IX**), berberrubine (**X**) and theophyline (**I.S**.) in the postive mode.

In present study, a sensitive and rapid UHPLC-ESI-MS/MS method was established to simultaneously quantify 10 alkaloids in beagle dog plasma. The alkaloids can be analysed by positive ESI mass spectrometry. Consequently, ESI operated in positive mode was preferentially selected as the ionization source for the compounds. Owing to the fact biological samples are complex, the MRM mode was selected to increase the specificity of the detection method. As Figure 1 shows, the intense peaks were observed at *m/z* 370.2→192.1 for corydaline (I), *m/z* 366.1→350.1 for dehydrocorydaline (II), *m/z* 356.0→192.0 for tetrahydropalmatine (III), *m/z* 354.1→188.0 for protopine (IV), *m/z* 352.1→336.2 for palmatine (V), *m/z* 340.1→176.1 for tetrahydroberberine (VI), *m/z* 339.2→323.2 for columbamine (VII), *m/z* 336.2→320.1 for berberine (VIII), *m/z* 320.2→292.2 for coptisine (IX), *m/z* 322.2→307.2 for berberrubine (X) and *m/z* 181.2→124.0 for theophyline (I.S.), respectively. The mobile phases consisting of 0.1% formic acid/water (A) and acetonitrile (B) could achieved a satisfactory separation effect. The flow rate was 0.3 mL/min and the column temperature was 35 °C Under the selected conditions, all compounds were separated rapidly in 10.5 min without interference from other components in beagle dog plasma.

### 2.2. Method Validation

#### 2.2.1. Selectivity

**Figure 2:**
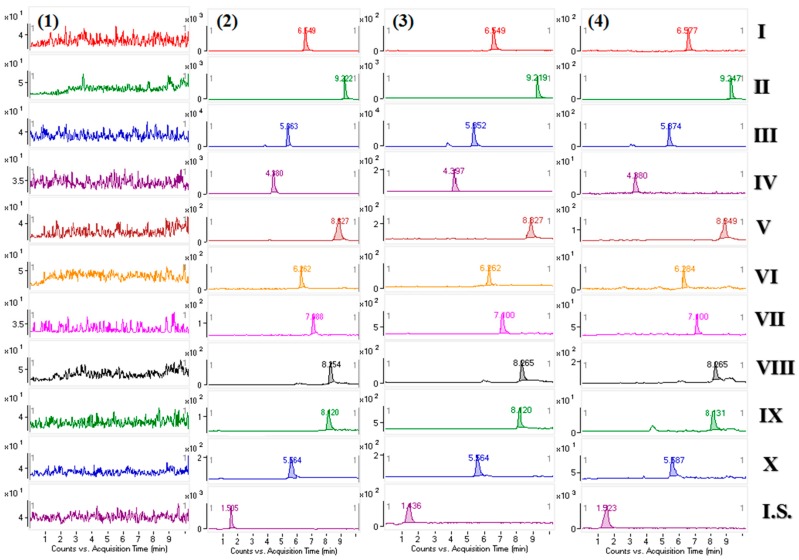
Representative MRM chromatograms of corydaline (**I**), dehydrocorydaline (**II**), tetrahydropalmatine (**III**), protopine (**IV**), palmatine (**V**), tetrahydroberberine (**VI**), columbamine (**VII**), berberine (**VIII**), berberrubine (**IX**), coptisine (**X**) and theophyline (**I.S.**) in the beagle dog plasma: (**1**) drug-free beagle dog plasma sample; (**2**) A beagle dog plasma sample taken 0.75 h after administration of 0.150 g/kg Yuanhuzhitong Tablets; (**3**) A beagle dog plasma sample taken 0.75 h after administration of 0.048 g/kg *C. yanhusuo* extraction; (**4**) LLOQ sample (10 alkaloids and I.S. in blank plasma).

The selectivity of the method was evaluated by analyzing drug-free beagle dog plasma sample, a beagle dog plasma sample taken at 0.75 h after administration of 0.150 g/kg Yuanhuzhitong Tablets, a beagle dog plasma sample taken at 0.75 h after oral administration of 0.048 g/kg *C. yanhusuo* extraction and LLOQ sample (10 alkaloids and I.S. spiked in blank plasma). The retention time of I-X was 6.57 min, 9.24 min, 5.67 min, 4.38 min, 8.84 min, 6.28 min, 7.10 min, 8.26 min, 8.13 min and 5.58 min, respectively. The retention time of I.S. was 1.52 min. The results are shown in Figure 2. In the selectivity test, there was no endogenous interference in the blank plasma samples at the mass transitions of the alkaloids at the same retention times.

#### 2.2.2. Linearity and LLOQs

Table 1 shows the regression equations, linear ranges, and LLOQ for the determination of the alkaloids in beagle dog plasma. All 10 alkaloids exhibited good linearity, and all *r*^2^ of the linear equations were higher than 0.9800. The linear calibration ranges were 0.54–557.0 ng/mL for I, 0.25–257.5 ng/mL for II, 0.50–507.0 ng/mL for III, 0.53–540.0 ng/mL for IV, 0.51–525.0 ng/mL for V, 0.41–420.0 ng/mL for VI, 0.39–400.0 ng/mL for VII, 0.53–540.0 ng/mL for VIII, 0.49–500.0 ng/mL for IX and 0.22–230.0 ng/mL for X, respectively. Accordingly, the concentrations can be determined by linear regression within the calibration range, which are sufficient for a pharmacokinetic study of 10 constituents following oral administration of *C. yanhusuo* and YHZT, respectively.

#### 2.2.3. Precision and Accuracy

The accuracy and precision experiments were performed with three levels of QCs (HQC, MQC, LQC) and LLOQ. The results indicated that the method was reliable and reproducible for the determination of beagle dog plasma samples. The results are listed in Table 2. The RSD of intra-day and inter-day precision were within 15%, and the accuracy within reasonable range. These results conformed to the requirements that defined by regulatory guidelines.

#### 2.2.4. Extraction Recovery and I.S.-Normalized Matrix Factor

The results of extraction recovery was shown in Table 3. The selected extraction solvent had a good extraction efficiency. The mean recoveries of 10 alkaloids were in the range of 79.09–98.55% at the concentrations of LQC, MQC, HQC samples and the mean recovery of the I.S. was 90.41 ± 6.24%. The IS-normalized matrix factor of QC samples were showed in Table 3. These results illustrated that the developed method had no matrix effect and was reliable for bioanalysis.

#### 2.2.5. Stability

The stability of the 10 alkaloids in beagle dog plasma was investigated under a variety of storage and process conditions. Table 4 summarizes the results of freeze and thaw, long-term, room temperature, and post-preparation stability experiments (The QC samples were kept in the auto-sampler at 4 °C for 12 h.). The measured concentrations were all within acceptable limits (±15% of the nominal concentrations) during the entire validation. The results of stability indicated that the 10 alkaloids were stable under conditions assessed in this method. 

### 2.3. Pharmacokinetic Studies

**Figure 3:**
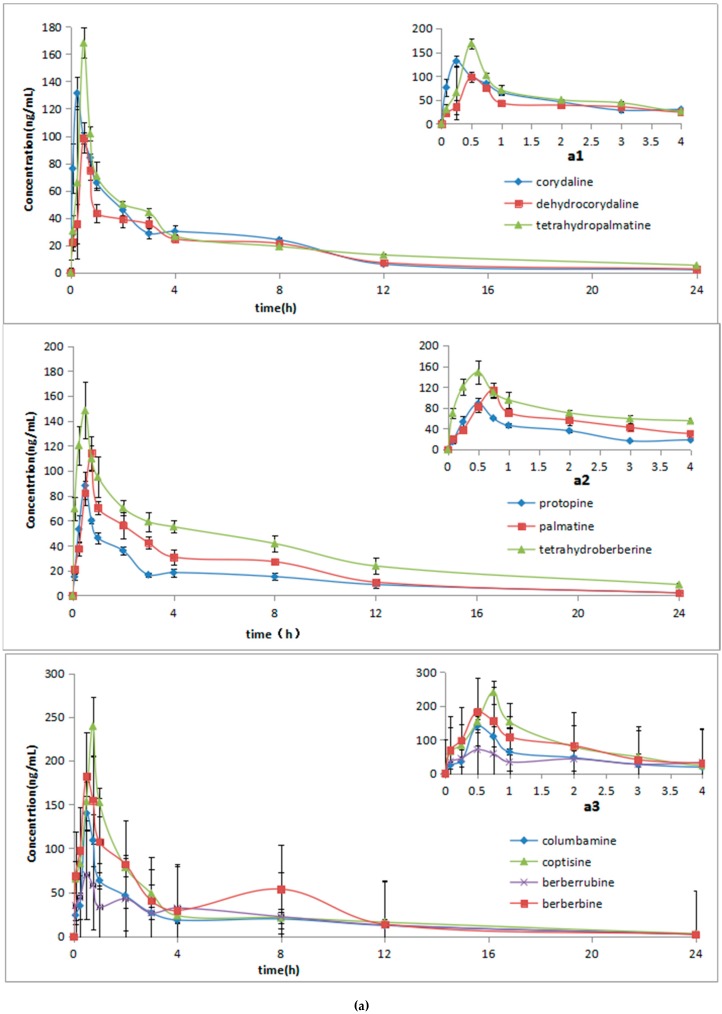

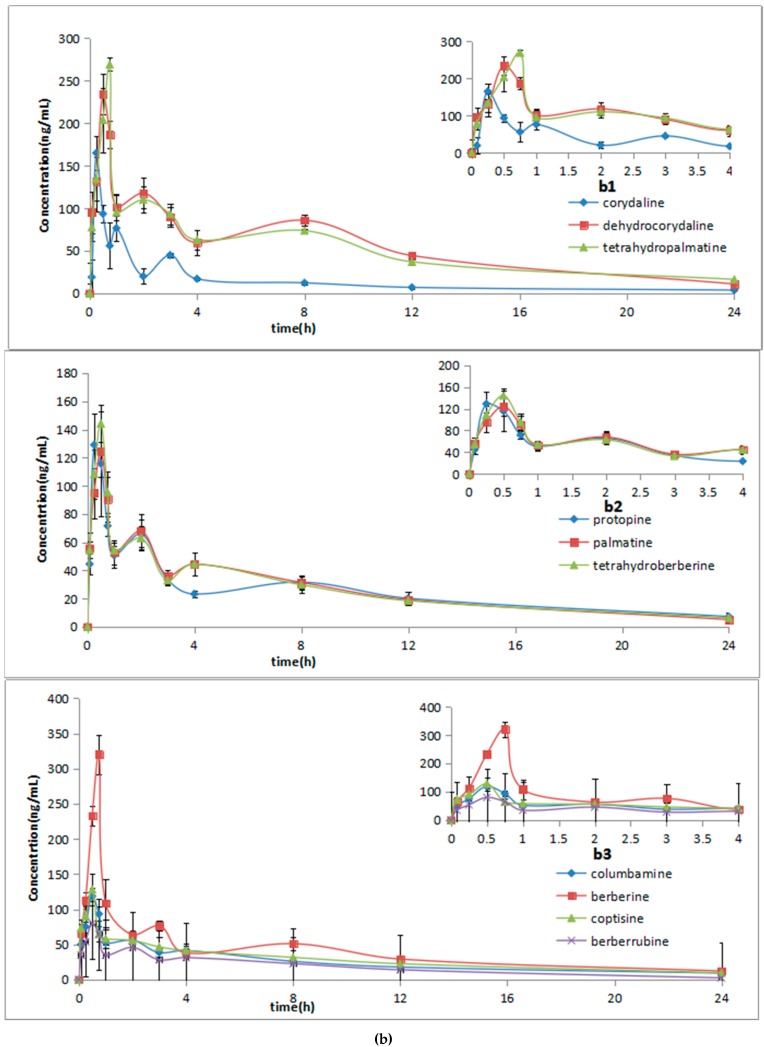
The mean ± SD plasma concentration-time profiles of the 10 alkaloids in the beagle dog plasma after oral administration of *C. yanhusuo* extract (**a**) and Yuanhuzhitong Tablets (**b**), respectively. The mean ± SD plasma concentration-time profiles of the 10 alkaloids in the beagle dog plasma after oral administration of *C. yanhusuo* extract (**a1**,**a2**,**a3**) and Yuanhuzhitong Tablets (**b1**,**b2**,**b3**) from 0 to 4 h, respectively (*n* = 6).

An UHPLC-ESI-MS/MS method for simultaneous determination of 10 alkaloids of *C. yanhusuo* extract and YHZT in beagle dog plasma was applied to the plasma sample analysis in the pharmacokinetic study. After oral administration, the main plasma concentration-time profiles of the 10 alkaloids were showed in Figure 3. Pharmacokinetic information of palmatine, tetrahydroberberine, columbamine, coptisine and berberrubine from *C. yanhusuo* extraction and YHZT were firstly obtained from beagle plasma. The concentrations versus time profiles were analyzed by a non-compartmental model. The results are listed in Table 5. The pharmacokinetic study indicated that the YHZT can get a better satisfactory absorbtion than *C. yanhusuo*.

## 3. Discussion

### 3.1. Selection of Extraction Method

Choosing an appropriate sample preparation method is the key to simultaneously and accurately analyzing the target compound [24]. Liquid–liquid extraction (LLE) and protein precipitation (PPT) are the most frequently used methods for sample preparations. PPT is evaluated for its convenient operation and high recovery rate, but it is not suitable for the low content of the analytes. Ethyl acetate, ether, dichloromethane and acetone were tested as the extraction solvent, the results showed that 3 mL ethyl acetate could achieve the aim of both high extraction recovery and weak matrix factor. The remaining extraction solvents could not achieve a satisfactory effect.

### 3.2. Selection of I.S.

It was critical to select an appropriate I.S. for the analysis of the whole biological samples. During the selection of I.S., biphenyl diester was found to possess a weak polarity and a long retention time under this mobile phase. The polarity of theophylline is similar to the alkaloid components, which could effectively shorten the analysis time. In addition, the result showed that theophylline had no direct interference in the analysis. 

### 3.3. Pharmacokinetic Studies

In this article, a new UHPLC-ESI-MS/MS method was developed for simultaneous determination of 10 alkaloids in beagle dog plasma after the oral administration of *C. yanhusuo* extraction and YHZT. YHZT is composed of two herbs including Radix Angelicae dahuricae and *Corydalis yanhusuo* W.T. Wang (vinegar-process). Through the comparison of the above two groups, the following conclusions can be obtained. Firstly, the *t_max_* of the two groups was shorter than 1h, which could be attributed that the active ingredient of *C. yanhusuo* and YHZT known as alkaloids belonging to quaternary ammonium alkaloids, are all dissolved easily in blood. It also showed that these alkaloids entered the blood through the absorption in stomach [30]. Secondly, we can see the *C_max_* of corydaline, dehydrocorydaline, tetrahydropalmatine, protopine, berberine and berberruine in YHZT group were higher remarkably than those in the *C. yanhusuo* group (*p* < 0.05). The explanation could be that the extraction rate of alkaloids in YHZT group was higher than that in *C. yanhusuo* group due to the processing method with vinegar as mentioned in *Introduction*. Thirdly, the AUC_0→__∞_ and AUC_0→t_ of dehydrocorydaline, tetrahydropalmatine, protopine, palmatine, columbamine and berberine in YHZT group are higher remarkably than that of the *C. yanhusuo* group (*p* < 0.05). It indicated that the bioavailability of YHZT group was higher than that of the *C. yanhusuo* group. Moreover, the *t*_1/2_ of corydaline, protopine, columbamine, berberine and coptisine in YHZT group is longer than these in the *C. yanhusuo* group. It indicated that the elimination time of YHZT is longer than *C. yanhusuo*. Thus, it had a guidance for clinical medication. Finally, following oral administration of *C. yanhusuo* extraction and YHZT, multiple-peaks phenomenon was observed on the mean plasma concentration curves of 10 analytes, and this phenomenon could be attributed to distribution, re-absorption and enterohepatic circulation [31]. 

## 4. Materials and Methods

### 4.1. Materials

Corydaline (151118), dehydrocorydaline (16051707), tetrahydropalmatine (151123), protopine (17062106), palmatine (140821), tetrahydroberberine (151111), columbamine (141108), berberine (141128), coptisine (140430), berberrubine (140407) and theophyline (17011102) with over 98% purity were purchased from Chengdu Pufei De Biotech Co. Ltd. (Chengdu, Sichuan Province, China). HPLC grade methanol and acetonitrile were purchased from Dikma Technologies Inc (Beijing, China). All other reagents including ethyl acetate, ether, dichloromethane and acetone belonged to analytical grade. Ultra-pure water was prepared by using a Milli-Q water purification system (Millipore, Molsheim, France). The blank plasma samples were achieved from the blood of healthy beagle dogs. Yuanhuzhitong tablet (20151201) was purchased from Sunflower Pharmaceutical Industry (Harbin, China). *Corydalis yanhusuo* W.T. Wang was obtained from the Panan Traditional Chinese Crude Drug Market, located in Zhejiang Province, China, and it was identified by Professor Zhenyue Wang of Heilongjiang University of Chinese Medicine in May, 2017.

### 4.2. UHPLC-ESI-MS/MS Conditions

The UHPLC-ESI-MS/MS was performed on an Agilent 1290 ultra-high performance liquid chromatography (UHPLC) system and an Agilent 6430 QQQ-MS mass spectrometer with an electrospray ionization (ESI) source interface (Agilent Technologies, Santa Clara, CA, USA). The UHPLC-ESI-MS/MS was performed on an Agilent Eclipse Plus C18 RRHD column (1.8 µm, 50 × 2.1 mm). In order to obtain the maximum sensitivity of the MRM, parameters such as fragmentor, collision energy and the nitrogen flow rate were optimized. The results showed in the Table 6. The mobile phases used 0.1% formic acid/water (A) and acetonitrile (B) as follows: 0–7.5 min, 22–32% (B); 7.5–8.5 min, 32–43% (B); 8.5–10.5 min, 43–55% (B). The flow rate was 0.3 mL/min. The column temperature was 35 °C. 5 μL sample solution was injected with a needle wash process. High-purity N_2_ was used as the nebulizing gas, and N_2_ was used as drying gas at a flow rate of 11 L/min. The mass spectrometer was operated in positive-ion mode at a capillary voltage of 4500 V. The source temperature was kept at 100 °C. The desolvation temperature was kept at 350 °C. 

### 4.3. Preparation of Standard and QC Solutions 

A stock standard solution with compound I–X mixing together was prepared in methanol with a concentration of 223.0, 206.0, 200.0, 113.0, 216.0, 200.0, 202.0, 201.0, 200.0 and 230.0 µg/mL, respectively. Theophyline (I.S.) was prepared with methanol at concentration of 1050.0 ng/mL. A series of calibration standards solutions were prepared at seven different concentration levels, I (557.0, 139.3, 34.8, 8.7, 2.2, 1.1 and 0.5 ng/mL), II (257.5, 64.4, 16.1, 4.0, 1.0, 0.5 and 0.3 ng/mL), III (507.0, 126.75, 31.68, 7.920, 1.980, 0.9902 and 0.4951 ng/mL ), IV (540.0, 135.0, 33.8, 8.5, 2.1, 1.1 and 0.5 ng/mL), V (525.0, 131.3, 32.8, 8.2, 2.1, 1.0 and 0.5 ng/mL), VI (420.0, 105.0, 26.3, 6.6, 1.6, 0.8 and 0.4 ng/mL), VII (400.0, 100.0, 25.0, 6.3, 1.6, 0.8 and 0.4 ng/mL), VIII (540.0, 135.0, 33.8, 8.4, 2.1, 1.1 and 0.5 ng/mL), IX (500.0, 125.0, 31.3, 7.8, 2.0, 1.0 and 0.5 ng/mL) and X (230.0, 57.5, 14.4, 3.6, 0.9, 0.4 and 0.2 ng/mL) respectively. Three different concentration levels of the QC samples were prepared for this assay, high QC (445.6/206.0/405.6/432.0/420.0/336.0/320.0/432.0/400.0/184.0 ng/mL), medium QC (34.8/16.1/31.7/33.8/32.8/26.3/25.0/33.8/31.3/14.4 ng/mL), low QC (1.1/0.5/1.0/1.1/1.0/0.9/0.8/1.0/1.0/0.4 ng/mL), and LLOQ (0.5/0.3/0.5/0.5/0.5/0.4/0.4/0.5/0.5/0.2 ng/mL), for I–X, respectively. All above samples were kept at 4 °C until use.

### 4.4. Preparation of C. yanhusuo Extract and YHZT

After soaking for 24 h, the *C. yanhusuo* was extracted twice with 60% ethanol (1:4, *w/v*) at 75 °C, 3 h for the first time and 2 h for the second time. The combined decoction was filtered and it was concentrated. Then, the paste rate is calculated. The contents of *C. yanhusuo* extract for I–X were 0.73, 1.50, 1.12, 0.92, 1.51, 1.03, 1.01, 0.81, 0.89 and 0.47 mg/g, respectively.

According to Chinese Pharmacopoeia, the dosage of *C. yanhusuo* medicinal materials for human is 10 g [1]. Specific surface area of human to dog equivalent dose ratio of body surface area conversion betwwen the human and dog is 0.32. The dosage of *C. yanhusuo* medicinal materials for dog is 0.27 g/kg. After a series of extraction and concentration operations, the extraction ratio of *C. yanhusuo* medicinal materials is 18%. Thus, the dosage of *C. yanhusuo* extract for beagle dog is 0.0486 g/kg.

The formulation illustration of YHZT was prescribed in the Chinese Pharmacopoeia [1]. 1000 Yuanhuzhitong tablets were made of 223 g of Radix Angelicae dahuricae and 445 g of vinegar-processed *Corydalis yanhusuo* W.T. Wang medicinal materials by a series of extraction and concentration operations. Each tablet was equivalent to 0.445 g *C.yanhusuo* medicinal materials. And according to Chinese Pharmacopoeia, the weight of the tablet core is 0.25 g [1]. Therefore, every gram YHZT powders was equivalent to 1.78 g *C.yanhusuo* medicinal materials. After taking off the Sugar coating, the tablets were lapped into powder. Then, the YHZT powder was weighed precisely. As mentioned above, the dosage of *C. yanhusuo* medicinal materials for beagle dog is 0.27 g/kg. It was equivalent to 0.15 g YHZT powders.

### 4.5. Biosample Preparation

A liquid-liquid extraction method was selected for the beagle dog plasma sample preparation. Fifty µL of internal standard solution (1050 ng/mL), 100 µL aliquot of plasma samples, and 100 µL of methanol were pipetted into a 10 mL glass tube. The mixture solution was vortex-mixed in glass tubes for 60 s. Then it was extracted with 3 mL ethyl acetate, vortex-mixed for 120 s, centrifuged at 877× *g* for 5 min. The organic layer was transferred into another spotless tube. Then, it was dried under a flow of nitrogen at 40 °C. The residue was re-dissolved by 100 µL mobile phase, vortex-mixed for 120 s, filtered by a 0.22 μm nylon 66 membrane. A 5 µL aliquot was injected into the UHPLC-ESI-MS/MS system.

The calibration standards and the QC samples were prepared under the same conditions as the plasma samples, which collected from the beagle dog after oral administration *C. yanhusuo* and YHZT powders. The spiked samples were handled by the following steps. 50 µL of internal standard solution (1050 ng/mL), 100 µL aliquot of blank plasma samples, and 100 µL of calibration standards or the QC methanol solution were pipetted into a 10 mL glass tube. And the mixture solution was vortex-mixed in glass tubes for 60 s. Then it was extracted with 3 mL ethyl acetate, vortex-mixed for 120 s, centrifuged at 877× *g* for 5 min. The organic layer was transferred into another spotless tube. Then, it was dried under a flow of nitrogen at 40 °C. The residue was re-dissolved by 100 µL mobile phase, vortex-mixed for 120 s, filtered by a 0.22 μm nylon66 membrane. The 5 µL samples was detected by the UHPLC-ESI-MS/MS system. 

### 4.6. Method Validation

Validation procedures were accomplished by the US Food and Drug Administration (FDA) guidelines for bioanalytical method validation, http://www.fda.gov/downloads/Drugs/GuidanceComplianceRegulatoryInformation/Guidances/UCM368107.pdf [32]. Parameters such as recovery, linearity, accuracy, precision, selectivity, matrix effect and stability of samples were investigated.

#### 4.6.1. Selectivity

In order to rule out the interference from endogenous matrix compounds with 10 analytes and I.S., method specificity was determined in beagle dog plasma. Six different blank plasma were obtained from different beagle dog plasma, and spiked with the alkaloids and I.S. at LLOQ level, then analyzed by UHPLC-MS/MS system. The response of co-eluting interferences was evaluated by the comparison of the drug free plasma chromatograms, the blank plasma chromatograms which spiked with analytes and I.S., and the plasma samples from the beagle dogs after oral administration of the *C. yanhusuo* extract and YHZT.

#### 4.6.2. Linearity and LLOQ

Each calibration curve was assessed by plotting the peak area ratio of the analytes and I.S. (*Y*) versus each nominal concentration (X) of the analytes. The calibration model was selected based on the analysis of the data by linear regression with weighting factors (1/x^2^). The LLOQ was determined as the lowest concentration point of the calibration curve with sufficient precision and the accuracy. The LLOQ could be quantified with S/N, which should be bigger than 10. Six replicate samples were used for evaluation.

#### 4.6.3. Precision and Accuracy

The precision and accuracy of the intra-day and inter-day were determined by the analysis of LLOQ, LQC, MQC and HQC samples (*n* = 6) on the one day and on three different days, respectively. Accuracy was evaluated as the relative error (RE) of the measured mean value. And the precision was determined as the RSD of the measured concentration. The concentration of each sample was calculated via a calibration curve that constructed on the same day. The criteria for precision and accuracy of evaluation are as follows: the accuracy should be less than 15% of the actual value for QC samples and the RSD should no more than 15%. The RSD of LLOQ samples should not exceed 20%.

#### 4.6.4. Extraction Recovery and I.S.-Normalized Matrix Factor

The extraction efficiency of the ten analytes was investigated by the comparison of six replicates beagle dog plasma samples at low, medium and high QC levels. The relative recoveries of the 10 analytes and the I.S. were determined by comparing the peak areas of an extracted sample against a post-extraction spiked sample and calculated by the ratio of the peak responses. The matrix factor was evaluated by comparing the absolute peak areas of blank matrix samples spiked after extraction with analytes to pure solution of the analytes. The I.S.-normalized matrix factor was calculated by the matrix factor ratio of the analyte to the internal standard.

#### 4.6.5. Stability

The stability test included three freeze-thaw cycles stability (−40 to 23 °C), room temperature stability (storage for 4 h at ambient temperature), long-term sample storage stability (−20 °C for two weeks) and ready-to-injection of extracted sample stability (4 °C for 12 h). LQC, MQC, HQC samples with five replicates at each level were kept at the above conditions and analyzed against freshly prepared calibrators as the reference.

### 4.7. Application to Pharmacokinetic Studies

Animal experimental procedures were carried out in accordance with the Guide for the Care and Use of Laboratory Animals, and the study was approved by the Animal Ethics Committee of the Institution. Twelve healthy male beagle dogs (body weight 12 ± 2 kg) were obtained from Shenyang Kangping Institute of Medical Laboratory Animals (SCXK (Liao) 2014-0003). Then it was randomly divided into two groups. All experimental procedures were approved by the ethics committees of Harbin Medical University. The blood samples of beagle dog were achieved at specific time points after oral administration of *C. yanhusuo* extraction with a dose of 0.0486 g/kg and YHZT powders with a dose of 0.15 g/kg. The beagle dogs were fasted overnight before experiment, and had free water supply even during the experiment. For animals, after oral administration of *C. yanhusuo* extraction and YHZT powders, 0.5 mL blood samples were withdrawn from the forearm vein into heparinized tubes at 0, 0.083, 0.25, 0.5, 0.75, 1.0, 2.0, 3.0, 4.0, 8.0, 12.0 and 24.0 h. Blood samples were centrifuged at 4582× *g* for 6 min and the upper layer plasma were kept at −20 °C until detection.

### 4.8. Data Analysis

In order to determine the pharmacokinetics parameters of 10 alkaloids, all data were processed by non-compartmental analysis using the DAS 2.0 software package (Chinese Pharmacological Society, Shanghai, China). The plasma concentration at different times was expressed as mean ± SD, and the mean concentration-time curves were plotted. The corresponding pharmacokinetic parameters (the elimination half-life, *t*_1/2_; area under the peak area-time curve from time zero to last sampling time AUC_0→t_ and AUC_0→__∞_) were tested. The elimination rate constant (*K_e_*) was calculated by linear regression of the terminal points in a semi-log plot of the plasma concentration against time. The elimination half-life was calculated using the formula *t*_1/2_ = 0.693/*K_e_*. The area under plasma concentration-time curve (AUC_0→t_) to the last measurable plasma concentration (*C_t_*) was estimated by using the linear trapezoidal rule. The area under the plasma concentration-time curve to time infinity (AUC_0→__∞_) was calculated as AUC_0→__∞_ = AUC_0→t_ + *C_t_*/*K_e_*. Moreover, the maximum observed plasma concentration (*C_max_*) and the time of first occurrence of *C_max_* (time for maximal concentration, *T_max_*) were also determined.

## 5. Conclusions

In the present article, a reliable and precise UHPLC-ESI-MS/MS method has been developed for the simultaneous quantification of 10 alkaloids in beagle dog plasma after oral administration of *C. yanhusuo* extraction and YHZT. This paper indicated that the YHZT could get a better absorbtion than *C. yanhusuo*. It will lay the foundation for the future study of *C. yanhusuo*.

## Figures and Tables

**Table 1:** The regression equations, linear ranges, and LLOQ for the determination of the alkaloids in the beagle dog plasma. (*n* = 7).

Compound	Regression Equation	*r* ^2^	Linear Range (ng/mL)	LLOQ (ng/mL)
corydaline	*Y* = 1.395 × 10^−3^*X* + 1.874 × 10^−3^	0.9938	0.54–557.0	0.54
dehydrocorydaline	*Y* = 9.668 × 10^−4^*X* + 3.035 × 10^−3^	0.9912	0.25–257.5	0.25
tetrahydropalmatine	*Y* = 1.368 × 10^−3^*X* − 2.608 × 10^−4^	0.9810	0.50–507.0	0.50
protopine	*Y* = 4.086 × 10^−4^*X* − 3.221 × 10^−5^	0.9870	0.53–540.0	0.53
palmatine	*Y* = 4.409 × 10^−4^*X* − 7.801 × 10^−5^	0.9868	0.51–525.0	0.51
tetrahydroberberine	*Y* = 6.810 × 10^−4^*X* + 1.381 × 10^−4^	0.9801	0.41–420.0	0.41
columbamine	*Y* = 4.941 × 10^-4^*X* − 1.679 × 10^−4^	0.9936	0.39–400.0	0.39
berberine	*Y* = 1.667 × 10^−3^*X* + 6.950 × 10^−3^	0.9888	0.53–540.0	0.53
coptisine	*Y* = 3.263 × 10^−4^*X* + 5.393 × 10^−3^	0.9934	0.49–500.0	0.49
berberrubine	*Y* = 1.623 × 10^2^*X −* 2.685 × 10^−3^	0.9816	0.22–230.0	0.22

**Table 2:** Precision and accuracy of the determination of the 10 alkaloids in the beagle dog plasma (*n* = 18, six replicates per day for three days).

Compound	Spiked Concentration (ng/mL)	Measured Concentration (ng/mL)	Accuracy (RE%)	Intra-Day Precision (RSD%)	Inter-Day Precision (RSD%)
corydaline	0.5	0.53 ± 0.03	−7.50	10.90	6.00
	1.1	0.96 ± 0.07	−10.75	8.22	2.53
	34.8	32.87 ± 1.69	−5.56	5.18	4.82
	445.6	432.80 ± 18.00	−2.86	3.99	5.24
dehydrocorydaline	0.3	0.21 ± 0.04	−12.10	12.10	12.40
	0.5	0.43 ± 0.05	−4.95	4.43	8.30
	16.1	14.59 ± 1.45	−9.34	9.94	10.13
	206.0	196.20 ± 8.40	−4.75	3.81	6.79
tetrahydropalmatine	0.5	0.45 ± 0.03	−6.90	8.80	12.10
	1.0	0.93 ± 0.05	−5.82	5.00	7.95
	31.7	30.04 ± 1.50	−5.18	5.19	2.72
	405.6	401.50 ± 20.26	−3.84	4.15	4.22
protopine	0.5	0.48 ± 0.02	−11.90	9.20	8.80
	1.1	0.94 ± 0.04	−5.96	4.97	1.53
	33.8	31.54 ± 0.88	−6.56	2.76	2.96
	432.0	403.30 ± 14.58	−6.64	3.61	3.64
palmatine	0.5	0.47 ± 0.02	−9.60	7.00	12.00
	1.0	0.91 ± 0.11	−12.32	10.51	7.09
	32.8	30.56 ± 1.48	−6.83	5.02	3.42
	420.0	401.90 ± 12.13	−4.29	2.94	3.54
tetrahydroberberine	0.4	0.39 ± 0.03	−12.90	7.79	7.30
	0.8	0.78 ± 0.06	−4.46	7.57	4.67
	26.3	24.17 ± 1.80	−7.92	7.66	5.50
	336.0	306.40 ± 24.86	−8.81	8.02	8.80
columbamine	0.4	0.38 ± 0.02	−10.50	11.40	13.70
	0.8	0.76 ± 0.04	−3.19	5.04	4.61
	25.0	24.02 ± 1.41	−3.92	6.19	2.35
	320.0	295.20 ± 7.17	−7.74	2.16	3.91
berberine	0.5	0.42 ± 0.05	−14.40	11.20	14.90
	1.1	0.87 ± 0.09	−13.22	10.66	3.28
	33.8	32.04 ± 0.74	−5.07	2.40	1.35
	432.0	411.60 ± 17.07	−4.73	4.22	3.60
coptisine	0.5	0.47 ± 0.04	−5.90	8.80	13.40
	1.0	0.92 ± 0.06	−7.71	6.13	7.29
	31.3	30.28 ± 0.89	−3.10	2.78	3.97
	400.0	389.40 ± 9.20	−2.66	2.30	2.78
berberrubine	0.2	0.18 ± 0.04	−16.09	19.40	18.20
	0.5	0.41 ± 0.02	−8.70	5.93	6.70
	14.4	13.48 ± 1.15	−6.18	7.89	12.47
	184.0	185.50 ± 10.41	0.83	5.15	8.30

**Table 3:** IS-normalized matrix factor and extraction recovery for the 10 alkaloids in the beagle dog plasma. (*n* = 6).

Compound	Spiked Concentration (ng/mL)	IS-Normalized Matrix Factor	Extraction Recovery	
			Mean (%)	RSD (%)
corydaline	1.1	0.91	79.36	9.903
	34.8	0.97	85.91	6.930
	445.6	1.00	79.11	9.320
dehydrocorydaline	0.5	1.00	84.12	8.781
	16.1	1.04	88.02	8.180
	206.0	1.14	86.84	5.280
tetrahydropalmatine	1.0	0.94	90.72	9.730
	31.7	0.98	87.25	12.68
	507.0	1.01	87.96	10.15
protopine	1.1	1.15	84.33	12.67
	33.8	1.08	91.22	13.75
	432.0	1.09	83.46	4.350
palmatine	1.0	0.94	91.21	11.01
	32.8	0.87	87.29	7.730
	420.0	0.74	84.82	7.230
tetrahydroberberine	0.8	0.89	89.17	12.30
	26.3	0.88	88.48	5.243
	336.0	0.92	93.00	8.870
columbamine	0.8	0.96	96.58	11.31
	25.0	0.91	91.61	11.58
	320.0	0.94	84.66	5.910
berberine	1.1	0.90	90.06	6.380
	33.8	0.94	84.58	5.520
	432.0	0.97	87.94	7.270
coptisine	1.0	0.95	91.75	9.640
	31.3	0.88	88.93	7.880
	400.0	0.95	85.60	6.890
berberrubine	0.5	0.94	88.41	7.790
	14.4	0.97	77.09	12.86
	184.0	0.91	98.55	5.000

**Table 4:** Stability data of 10 alkaloids in the beagle dog plasma. (*n* = 6).

Compound	Spiked Concentration (ng/mL)	Stability (% RE ^a^)			
		Short-Term	Long-Term	Three Freeze-Thaw	Post-Preparation
corydaline	1.1	3.86	4.90	−2.72	2.30
	34.8	−4.30	−4.52	−4.83	−4.36
	445.6	−1.88	−4.45	−3.63	−4.56
dehydrocorydaline	0.5	8.52	2.85	3.90	3.87
	16.1	4.15	5.16	10.41	13.40
	206.0	3.50	2.97	1.85	1.05
tetrahydropalmatine	1.0	−4.16	−4.47	−4.77	−4.36
	31.7	−1.92	−4.85	−4.76	2.09
	507.0	−2.69	−3.38	−3.36	−4.54
protopine	1.1	−4.47	−4.48	−6.91	−4.40
	33.8	−2.86	−4.78	−3.96	−3.63
	432.0	−4.96	−4.93	−4.38	−3.92
palmatine	1.0	−4.06	2.25	−2.08	−4.98
	32.8	−3.35	−4.59	−3.35	−3.35
	420.0	−3.83	−3.95	−2.02	−2.78
tetrahydroberberine	0.8	−2.54	−2.59	−4.69	−3.54
	26.3	−3.38	−4.95	−3.22	−4.74
	336.0	−3.32	−3.04	−3.92	−4.51
columbamine	0.8	−3.44	−3.46	−3.06	−1.75
	25.0	−2.54	−3.94	−4.49	−3.14
	320.0	−3.99	−4.88	−4.29	−4.42
berberine	1.1	−4.14	−4.51	−3.91	−3.47
	33.8	−1.87	−4.50	−4.62	−4.47
	432.0	−2.88	−4.28	−3.62	−4.08
coptisine	1.0	−5.42	−4.76	−3.90	−1.88
	31.3	−3.34	−4.31	−1.37	−4.31
	400.0	−4.14	−2.34	−2.09	−4.09
berberrubine	0.5	−12.10	−6.88	−7.75	−11.52
	14.4	10.68	−3.02	−13.61	−5.61
	184.0	1.39	−2.87	1.67	0.89

**^a^** RE is expressed as (measured concentration/freshly prepared concentration^−1^) × 100%.

**Table 5:** Pharmacokinetic parameters of the 10 alkaloids in the beagle dog after oral administration of *C. yanhusuo* (A) and YHZT (B), respectively. (mean ± SD, *n* = 6).

	Compounds	Cmax (ng/mL)	*T_max_* (h)	*t*_1/2_ (h)	AUC_0__→t_ (ng·h/mL)	AUC_0→∞_ (ng·h/mL)
(A)	corydaline	138 ± 24.5	0.29 ± 0.10	4.90 ± 0.70	358.34 ± 39.02	401.61 ± 48.38
	dehydrocorydaline	99.7 ± 12.3	0.54 ± 0.10	5.83 ± 1.75	342.28 ± 9.30	403.19 ± 30.18
	tetrahydropalmatine	169 ± 55.3	0.54 ± 0.10	9.09 ± 2.17	424.21 ± 20.94	591.20 ± 50.58
	protopine	89.4 ± 8.49	0.95 ± 0.13	5.75 ± 0.81	275.69 ± 18.43	349.18 ± 38.33
	palmatine	116 ± 13.9	0.95 ± 051	4.45 ± 0.71	397.05 ± 30.16	465.14 ± 34.69
	tetrahydroberberine	154 ± 24.9	0.50 ± 0.16	7.06 ± 1.35	711.63 ± 56.12	1044.40 ± 68.18
	columbamine	144 ± 11.01	0.54 ± 0.10	5.67 ± 1.95	404.21 ± 15.57	506.82 ± 40.75
	berberine	187 ± 23.4	0.54 ± 0.10	5.00 ± 0.90	593.56 ± 23.76	673.64 ± 29.62
	coptisine	242 ± 58.2	0.79 ± 0.10	5.89 ± 1.07	631.90 ± 86.03	748.34 ± 68.39
	berberrubine	72.1 ± 1.9	0.54 ± 0.10	5.01 ± 0.61	422.88 ± 23.26	440.26 ± 19.33
(B)	corydaline	165 ± 21.3 *	0.29 ± 0.10	11.24 ± 1.49	361.474 ± 43.83	435.90 ± 58.28
	dehydrocorydaline	187 ± 23.8 *	0.54 ± 0.10	5.61 ± 1.05	1306.88 ± 148.77 *	1399.25 ± 142.40 *
	tetrahydropalmatine	277 ± 23.7 *	0.71 ± 0.10	9.34 ± 0.26	1268.11 ± 61.64 *	1462.37 ± 98.46 *
	protopine	139 ± 24.2 *	0.38 ± 0.14	7.77 ± 0.81	601.27 ± 66.95 *	683.25 ± 67.68 *
	palmatine	127 ± 14.6	0.50 ± 0.16	6.09 ± 0.66	638.87 ± 43.52 *	684.56± 60.57 *
	tetrahydroberberine	145 ± 12.0	0.54 ± 0.10	7.65 ± 1.14	637.16 ± 59.08	718.80 ± 98.29
	columbamine	120 ± 83 *	0.54 ± 0.10	11.73 ± 2.92	611.24 ± 53.13 *	755.66 ± 95.47 *
	berberine	± 28.0 *	0.71 ± 0.10	8.48 ± 1.92	988.42 ± 116.18 *	1140.97 ± 91.92 *
	coptisine	133 ± 15.1 *	0.46 ± 0.10	9.58 ± 1.81	685.60 ± 60.34	824.82 ± 106.82
	berberrubine	86.0 ± 6.7 *	0.54 ± 0.10	5.05 ± 0.48	445.44 ± 27.44	464.11 ± 25.40

The Cmax value, AUC_0→t_ and AUC_0→__∞_ compared with *C. yanhusuo* group * *p* < 0.05.

**Table 6:** Quantitative, Qualifier ions and MS parameters of eight alkaloids and I.S.

Compound	Ion Pair (*m/z*)	Qualifier Ion (*m/z*)	Fragment (V)	CE (V)	Polarity
corydaline	370.2→192.1	165.1	170	30	Positive
dehydrocorydaline	366.1→350.1	334.1	140	30	Positive
tetrahydropalmatine	356.0→192.0	165.0	159	27	Positive
protopine	354.1→188.0	149.0	170	30	Positive
palmatine	352.1→336.2	294.1	155	40	Positive
tetrahydroberberine	340.1→176.1	149.0	168	40	Positive
columbamine	339.2→323.2	295.1	160	29	Positive
berberine	336.2→320.1	292.2	136	30	Positive
coptisine	320.2→292.2	292.2	167	29	Positive
berberrubine	322.2→307.2	250.1	160	29	Positive
theophyline (I.S.)	181.2→124.0	108.9	120	14	Positive

The cell accelerator volltage value of the compounds are 5V.
